# Macroporous Resin Recovery of Antioxidant Polyphenol Compounds from Red Onion (*Allium cepa* L.) Peel

**DOI:** 10.3390/antiox14020145

**Published:** 2025-01-26

**Authors:** Khanafina Aliya, Ha-Seong Cho, Ibukunoluwa Fola Olawuyi, Ju-Hwi Park, Ju-Ock Nam, Won-Young Lee

**Affiliations:** 1 School of Food Science and Technology, Kyungpook National University, Daegu 41566, Republic of Korea; khanafinaaliya@gmail.com (K.A.); hasung31694@knu.ac.kr (H.-S.C.); ifolawuyi@knu.ac.kr (I.F.O.); qkrwn9809@gmail.com (J.-H.P.); namjo@knu.ac.kr (J.-O.N.); 2 Research Institute of Tailored Food Technology, Kyungpook National University, Daegu 41566, Republic of Korea

**Keywords:** red onion peel, polyphenol compounds, macroporous resin, recovery, antioxidant and anti-inflammatory activities

## Abstract

In this study, polyphenols in the crude extract (CE) from red onion peel were recovered by macroporous resin, and their antioxidant and anti-inflammatory activities were evaluated. Among the four resins screened (SP850, XAD2, XAD7HP, and XAD16N), XAD7HP showed the highest desorption and recovery ratios, and it was used to optimize polyphenol recovery through single-factor experiments. The optimal conditions were established as 1 g resin, pH 4, 25 °C, 7 h for adsorption, followed by desorption with 70% ethanol for 1 h at 25 °C. These conditions achieved 85.00% adsorption ratio, 87.10% desorption ratio, and 20.9% yield of the macroporous resin-recovered extract (MRE) from the CE. HPLC analysis revealed that rosmarinic acid, quercetin, and myricetin were major compounds in the MRE, with the content of these compounds higher (about 7-fold) compared to the CE, confirming enhanced recovery of polyphenols by macroporous resin. Moreover, FT-IR and ¹H-NMR analysis confirmed the successful recovery of these polyphenol compounds in the MRE. Furthermore, the MRE displayed significantly improved antioxidant activities (DPPH, ABTS, and FRAP) and anti-inflammatory activities (inhibition of nitric oxide synthesis and reactive oxygen species production) compared to the CE. In summary, our findings suggest that macroporous resin can effectively recover polyphenol compounds from red onion peel extract and enhance their biological activities.

## 1. Introduction

Onion (*Allium cepa* L.) is one of the most commonly cultivated vegetable crops and ranks as the second most developed agricultural crop in the world [1]. Onion is typically consumed as the bulb and peels often thrown out as a by-product of industrial processing or cooking. However, onion peels are a richer source of bioactive compounds, particularly polyphenols, compared to the bulbs [2,3,4]. Among varieties of onion peels, red onion peel has been reported to possess superior antioxidant and free radical scavenging properties in vitro compared to purple, white, and green onion peels [3,5]. Moreover, the bioactive compounds in red onion peel have demonstrated significant biological effects, including anti-inflammatory, anti-obesity, anti-diabetic, cardiovascular, and anticancer properties [6,7]. Given these benefits, red onion peel has potential application as a natural resource in the food, pharmaceutical, and cosmetic industries.

Several extraction technologies, including enzyme-assisted, ultrasound-assisted, and supercritical fluid extraction, have been employed to extract polyphenol compounds from red onion peel [8,9]. In addition, diverse organic solvents, ethanol, toluene, and dichloromethane have been utilized. While these methods and solvents can effectively extract polyphenolic compounds from red onion peel, they are not selective and the extract may contain impurities, resulting in low polyphenol content [10,11]. Therefore, an additional process is required to facilitate the selective recovery of polyphenols from the crude extract, thus improving the biological activity of the purified extract.

Many recovery methods have been conducted to selectively enrich polyphenol compounds, including high-performance liquid chromatography [12], solid-phase extraction [13], and liquid-liquid extraction [14]. However, these techniques revealed a number of drawbacks, including low capacity, poor yield, and preparation of special apparatus [15]. By contrast, the macroporous resin recovery method is a widely used technique based on the adsorption-desorption process, with several advantages, such as low costs, simple procedures, and safety [16]. This approach was successfully conducted to the recovery of polyphenol compounds from a variety of sources like *Macrocystis pyrifera* [17,18], *Ecklonia cava* [18], and *Rabdosia serra* [19]. Additionally, several studies have reported that the macroporous resin recovery process enhanced the physiological properties of the extract, especially its antioxidant and anti-inflammatory effects [20,21,22].

Polyphenol compounds in aqueous solutions are primarily adsorbed onto the resin via hydrophobic interactions and aromatic stacking mechanisms. Following adsorption, an organic solvent is used to desorb the compounds, resulting an enriched target compound [17]. However, several factors, including the physical properties of the resin, resin type, and adsorption and desorption conditions, significantly affect the final recovery ratio, which determines the efficiency of the recovery process [23,24,25]. These studies confirmed that the various parameters, such as specific surface area, pore diameter, amount of resin, time, pH, and concentration, influence the recovery ratio. Nevertheless, there is no research on the adsorption-desorption process using the resin method to recover polyphenols from red onion peel and assess their antioxidant and anti-inflammation activities.

Therefore, this study was designed to screen different macroporous resins to identify the most effective resin for recovering polyphenolic compounds from red onion peel extract. In addition, the various adsorption parameters (resin amount, pH, temperature, adsorption time) and desorption conditions (ethanol concentration and desorption time) were investigated to optimize the recovery process using macroporous resin. Furthermore, the polyphenols composition (HPLC), structural characteristics (FT-IR and ^1^H-NMR), antioxidant properties (DPPH, ABTS, and FRAP scavenging), and anti-inflammatory activities (nitric oxide synthesis and reactive oxygen species production) were analyzed in the CE and MRE.

## 2. Materials and Methods

### 2.1. Materials and Chemical Reagents

Red onions (*Allium cepa* L.), ‘*Hongbanjang*’ variety were purchased in the autumn of 2024 from a local farm in Yeoju-si, Gyeonggi-do, Republic of Korea (37.36° N 127.59° E). The onions were peeled and dried in a dry oven (JSOF-100, Gongju-Si, Republic of Korea) for 24 h at 40 °C, and after were ground using a grinder (RT-04, Mill powder, Tainan, Taiwan) and kept in sealed vacuum bags at −18 °C for further experiments. Macroporous resins (SP850, XAD2, XAD7HP, and XAD16N), 2,2′-azinobis (3-ethylbenzothiazoline-6-sulfonic acid) (ABTS), 2,2-diphenyl-1-picrylhydrazyl (DPPH), and Folin–Ciocalteu’s reagent were ordered from Sigma–Aldrich Co. (St. Louis, MO, USA). The physical characteristics of the resins are shown in Table 1. All other analytical-grade reagents were purchased from Duksan Chemical Company (Ansan, Republic of Korea).

### 2.2. Preparation of Crude Extract (CE)

The onion peel extract was prepared following the method [26] with slight modifications. Briefly, dried red onion peel powder (5 g) was combined with 60% ethanol (100 mL) and stirred at 30 °C, 200 rpm for 60 min using a water bath shaker (Daihan Scientific Co., Ltd., Wonju, Republic of Korea). To remove solids, the resulting mixture was filtered through Whatman No. 1 filter paper (Whatman Int., Ltd., Maidstone, UK). After filtration, the ethanol in the supernatant was removed by evaporation at 45 °C using a vacuum evaporator (R-205V, BÜCHI Labortechnik AG, Flawil, Switzerland). Lastly, to obtain the CE powder form, the concentrated solution was lyophilized using a freeze dryer (FDS8518, Ilsin BioBase Co., Ltd., Dongducheon-si, Republic of Korea). The obtained powder was kept in a ziplock bag at −18 °C for further analysis.

### 2.3. Total Phenolic Content (TPC)

With slight modifications, the Folin–Ciocalteu method was employed to assess the total polyphenol content [27]. Briefly, the sample was dissolved at a concentration of 5 mg/mL in distilled water. A 0.1 mL aliquot of the sample was mixed with 0.05 mL of 2N Folin–Ciocalteu reagent and 0.3 mL of 2% Na_2_CO_3_ solution. The mixture was incubated at room temperature for 15 min, and then 1 mL of distilled water was added. The absorbance was measured at 725 nm using a Shimadzu UV-2550 UV-visible spectrophotometer (Tokyo, Japan). To determine the concentration of TPC (mg/mL) in the sample, a calibration curve was created using gallic acid as a standard. Gallic acid was dissolved in distilled water, with concentrations ranging from 0.025 to 0.25 mg/mL. The equation was y = 5.6605x − 0.0321 (R^2^ = 0.9987).

### 2.4. Static Adsorption and Desorption

The adsorption and desorption process were conducted with slight modifications to the method described by [18]. First, 1 g of macroporous resin was pretreated by soaking in 95% ethanol for 24 h at 25 °C in a shaking incubator (SI-600R, Jeio-tech, Seoul, Republic of Korea) at 200 rpm. Following pretreatment, the resin was placed in a 250 mL conical flask containing 50 mL of the CE solution (5 mg/mL), and it placed in a shaking incubator for adsorption at 25 °C for 24 h (200 rpm). After adsorption, the solution was removed and the resin was washed exhaustively with distilled water. Subsequently, the washed resin was then mixed with 50 mL of 70% ethanol, and the desorption was carried out by shaking at 25 °C and 200 rpm for another 24 h. The adsorption ratio, desorption ratio, and recovery ratio were calculated using the following equations:

(1) $$Adsorption\;ratio\;(\%)=\frac{\left(C_{0}-C_{e}\right)}{C_{0}}\times 100$$

(2) $$Desorption\;ratio\;(\%)=\frac{C_{d}}{\left(C_{0}-C_{e}\right)}\times 100$$

(3) $$Recovery\;ratio\;(\%)=\frac{C_{d}}{C_{0}}\times 100$$

where C_0_, C_e_, and C_d_ are the total phenolic contents (TPC) in the initial, adsorption, and desorption solutions (mg/mL), respectively.

### 2.5. Single-Factor Experiments

After selecting the best resin for the recovery of polyphenol compounds from the CE, single-factor experiments with the resin were conducted to optimize the adsorption and desorption conditions. For the optimal adsorption ratio, the effects of different resin amounts (0.5 g, 1 g, 1.5 g, and 2 g), pH levels of the extract solution (2, 4, 7, and 9), temperatures (15 °C, 25 °C, 35 °C, and 45 °C), and time range from 1 h to 24 h were evaluated. The pH of the CE solution was adjusted using 0.3 M HCl or 0.3 M NaOH. For the different times, 500 µL of the aliquot was taken as the fixed intervals. Once the optimal adsorption conditions were determined, desorption optimization experiments were conducted. The effect of ethanol concentration (30%, 50%, 70%, and 90%) and time range (from 1 h to 24 h), and temperature (15 °C, 25 °C, 35 °C, and 45 °C) on the desorption efficiency were investigated. For the desorption time, 500 µL of the sample was collected at certain times for the desorption time. The adsorption and desorption ratios were calculated according to the TPC content and Equations (1) and (2), respectively.

### 2.6. Adsorption Kinetics

To study the adsorption process, 1 g of pretreated resin was added to conical flasks containing 50 mL of extract solution (5 mg/mL) and placed in a shaking incubator at 200 rpm and 25 °C. Aliquots of 500 µL were collected at predetermined time intervals during adsorption, and the TPC was determined as described in Section 2.3. The adsorption kinetics were analyzed by fitting the experimental data to two mathematical models: the pseudo-first-order model (4) and the pseudo-second-order model (5).

(4) $${ln(Q}_{e}{-Q}_{t})=lnQ_{e}-K_{1}t$$

(5) $$\frac{1}{Q_{t}}=\frac{1}{K_{2}Q_{e}^{2}}\frac{1}{t}+\frac{1}{Q_{e}}$$

where Q_e_ is the adsorption capacity at equilibrium (mg/g); Q_t_ is the adsorption rate of phenolic compounds (mg/g) at time t (min); K_1_ (min^−1^) and K_2_ [g/(mg·min)] are the rate constants of the pseudo-first-order and pseudo-second-order models, respectively.

### 2.7. Determination of Desorption Activation Energy

The activation energy (E_d_) for the desorption process was calculated using the Arrhenius equation:

(6) $$k=Ae^{-\frac{E_{d}}{RT}}$$

where *E_d_* is the activation energy of desorption (J/mol), *k* is the desorption rate constant (min^−1^), A is the frequency factor (min^−1^), R is the universal gas constant (8.314 J/mol·K) and T is the absolute temperature (K).

### 2.8. HPLC Analysis

The HPLC method, as previously described by [28], was used to identify and quantify polyphenol compounds in the CE and MRE. All samples were prepared at a concentration of 1 mg/mL in 70% ethanol, followed by filtration through a 0.45 μm membrane filter. HPLC analysis was conducted using a UV/VIS HPLC system (JASCO International Co., Ltd., Tokyo, Japan), which included a UV-2075 plus detector (JASCO International Co., Ltd.) and an Athena C18 reversed-phase column (250 × 4.6 mm, 5 μm). The mobile phase consisted of 1% aqueous acetic acid (A) and acetonitrile (B), with gradient elution conditions as follows: 0–10 min at 10% B, 10–28 min with a linear gradient from 10% to 40% B, 28–39 min from 40% to 60% B, and 39–60 min with a gradient from 60% to 90% B. The flow rate was set at 0.7 mL/min, and the column temperature was set to 25 °C, respectively. UV detection was carried out at 254 nm with an injection volume of 20 μL. The quantification of the identified polyphenol compounds in the samples was performed using calibration curves constructed from standard solutions of each pure compound.

### 2.9. FT-IR and NMR Spectroscopy

The Fourier transform infrared (FT-IR) spectra of the CE and MRE were detected using a Frontier FT-IR Spectrophotometer (Perkin Elmer, Hopkinton, MA, USA). Samples (2 mg) were detected with a resolution of 4 cm^−1^ for a frequency range of 4000–400 cm^−1^.

The Nuclear magnetic resonance (NMR) experiments were conducted using a Bruker spectrometer (Billerica, MA, USA) equipped with a 5 mm probe at 298 K. Samples were dissolved in DMSO-d_6_ at a concentration of 1 mg/mL, and the ¹H-NMR spectra were acquired over 64 scans for the CE and MRE, and 32 scans for standard compounds at a temperature of 25 °C. The resulting spectra were processed and analyzed using MestReNova software (version 14.1.2, Mestrelab Research, Santiago de Compostela, Spain).

### 2.10. Antioxidant Activity

#### 2.10.1. ABTS Assay

ABTS scavenging activity was determined according to a method described by [29]. Different concentrations (50–500 μg/mL) of the CE and MRE were dissolved in distilled water. The ABTS cation radical was prepared by mixing 7 mM ABTS and 2.45 mM aqueous potassium persulfate in 10 mL of distilled water, and then by allowing the mixture to react in the dark for 16 h using a magnetic stirrer (PC-420D, Corning Co., Corning, NY, USA). Subsequently, a 0.05 mL aliquot of the CE and MRE was mixed with 0.95 mL of the ABTS solution and incubated in the dark for 30 min at room temperature. A UV spectrophotometer was then used to measure the absorbance at 734 nm. The scavenging activity of ABTS was calculated using the following equation:

(7) $$ABTS\left(\%\right)=(1-\frac{A_{1}}{A_{0}})\times 100$$

where A_1_ was the absorbance value for the sample, and A_0_ was the absorbance value for the control.

#### 2.10.2. DPPH Assay

DPPH was assessed using the method previously described by [30]. Different concentrations (50–500 μg/mL) of the CE and MRE were dissolved in distilled water. DPPH solution was prepared by dissolving 3.9 mg of 2,2-diphenyl-1-picrylhydrazyl in 100 mL of 95%, followed by stirring for 2 h using a magnetic stirrer. A 0.9 mL of DPPH solution was mixed with 0.1 mL aliquots of each sample, and the mixture was kept in the dark at room temperature for 30 min. Thereafter, the absorbance was then measured using a UV spectrophotometer at 517 nm. DPPH was measured using the following equation:

(8) $$DPPH\left(\%\right)=\left(1-\frac{A_{1}}{A_{0}}\right)\times 100$$

where A_1_ was the absorbance value for the sample, and A_0_ was the absorbance value for the control.

#### 2.10.3. FRAP Assay

The ferric reduction power (FRAP) was measured using a method [31]. Different concentrations (25–200 μg/mL) of the CE and MRE were dissolved in distilled water. A total of 0.1 mL aliquots of each sample were mixed with 0.9 mL of FRAP reagent and incubated in the dark at room temperature for 30 min. Absorbance was measured at 593 nm using a UV spectrophotometer. Results were expressed as micrograms of ascorbic acid equivalents per gram of sample (μg AAE/g).

### 2.11. Cell Culture and Cell Viability Analysis

Murine macrophage cell line RAW264.7 cell was obtained from the Korea Cell Line Bank (Seoul, Republic of Korea) and cultured in Dulbecco’s Modified Eagle Medium (Gibco, Grand Island, NY, USA) containing 10% fetal bovine serum (FBS, Gibco) and 1% penicillin/streptomycin. The cells were incubated in a humidified incubator of 37 °C and 5% CO_2_.

In accordance with the supplier’s instructions, cell viability was carried out using the cell counting kit-8 (CCK-8) (Dojindo, Kumamoto, Japan) assay. RAW264.7 cells were treated with or without varying concentrations (25, 50, 100 μg/mL) of the CE or MRE for 24 h. Next, the RAW264.7 cells were incubated with 10 μL of CCK-8 reagent (Dojindo, Kumamoto, Japan) for 2 h. The absorbance at 450 nm was observed applying an ELISA reader (Infinite f50, TECAN, Männedorf, Switzerland). The cell viability was calculated as follows:

(9) $$Cellviability\left(\%\right)=\frac{Absorbance\;of\;experimental\;sample}{Absorbance\;of\;negative\;control(NC)}\times 100$$

### 2.12. Assessment of Nitric Oxide (NO) Synthesis

The intracellular ROS production in RAW264.7 cells was measured using the 2′,7′-dichlorofluorescein diacetate (DCF-DA) assay. RAW264.7 cells (2 × 10^5^ cells/mL) were seeded in 6-well plates and pretreated with various concentrations (25, 50, 100 μg/mL) of the CE or MRE for 1 h then cotreated with LPS (1 μg/mL) for 24 h. Then, 20 μM of DCF-DA was added and incubated for 30 min. Subsequently, DCF-DA+ area was measured using flow cytometry (Thermo Fisher Scientific, Waltham, MA, USA), with an excitation at 488 nm and an emission at 530 nm.

### 2.13. DCF-DA Assay

2′,7′-dichlorofluorescein diacetate (DCF-DA) assay was performed to detect intracellular ROS production in RAW264.7 cells. RAW264.7 cells (2 × 10^5^ cells/mL) were seeded in 6-well plates and pretreated with various concentrations (25, 50, 100 μg/mL) of the CE or the MRE for 1 h and then cotreated with LPS (1 μg/mL) for 24 h. Then, 20 μM of DCF-DA was added and incubated for 30 min. Subsequently, DCF-DA+ area was measured using flow cytometry (Thermo Fisher Scientific, Waltham, MA, USA), at an excitation wavelength of 488 nm and an emission wavelength of 530 nm.

### 2.14. Statistical Analysis

All experiments, except FT-IR and ^1^H-NMR, were performed in triplicate. The data were analyzed using ANOVA with Duncan’s multiple range test (*p* < 0.05) using the IBM SPSS Statistic 25 program (SPSS Inc., Chicago, IL, USA).

## 3. Results and Discussion

### 3.1. Resin Screening

In the screening stage, four different macroporous resins (SP850, XAD7HP, XAD16N, and XAD2) were screened to determine suitable resin for polyphenol recovery from the CE. The adsorption, desorption, and recovery ratios are summarized in Table 2. As a result, SP850 showed the highest adsorption ratio (82.39 ± 0.95%), followed by XAD16N (82.15 ± 0.24%), XAD7HP (81.34 ± 0.24%), and XAD2 (76.20 ± 0.24%). Based on the physical properties of the resins (Table 1), SP850, XAD16N, and XAD2 are non-polar resins made from styrene-divinylbenzene; however, SP850 and XAD16N have larger surface areas compared to XAD2, enhancing interactions between resin and polyphenol compounds, resulting in more efficient adsorption [18]. Additionally, high adsorption capacity for the resins with large surface areas has also been reported for polyphenolic compounds from C. speciosa fruit [32]. On another note, XAD7HP has a lower surface area compared to other resins such as SP850 and XAD16N, but its adsorption ratio is comparable to these resins (*p* > 0.05), which could be due to its larger pore size [33]. Meanwhile, the desorption ratio of XAD7HP (78.54 ± 1.35%) was higher than that of SP850 (73.16 ± 1.18%), XAD16N (72.34 ± 1.75%), and XAD2 (65.69 ± 7.8%). This trend is in line with the previous study by Hou and Zhang [10] which reported that resins with a larger pore size demonstrated superior desorption capacity. The desorption ratio of the polyphenol compound is proportional to the resin pore size, which could be explained by the larger pore size facilitating easier elution of the adsorbate by the desorption agent [10]. Consequently, the recovery ratio of XAD7HP (67.03 ± 0.9%) was the highest among the tested resins. Therefore, XAD7HP was chosen as the most effective resin for the recovery of polyphenols from the CE. Furthermore, other studies also reported the effectiveness of XAD7HP resin in recovering polyphenolic compounds from other sources, including Vaccinium spp. [34], Rabdosia serra [19], and *Ipomoea batatas* L. [35].

### 3.2. Single-Factor Experiment on the Adsorption and Desorption

#### 3.2.1. Effect of Resin Amounts

**Figure 1:**
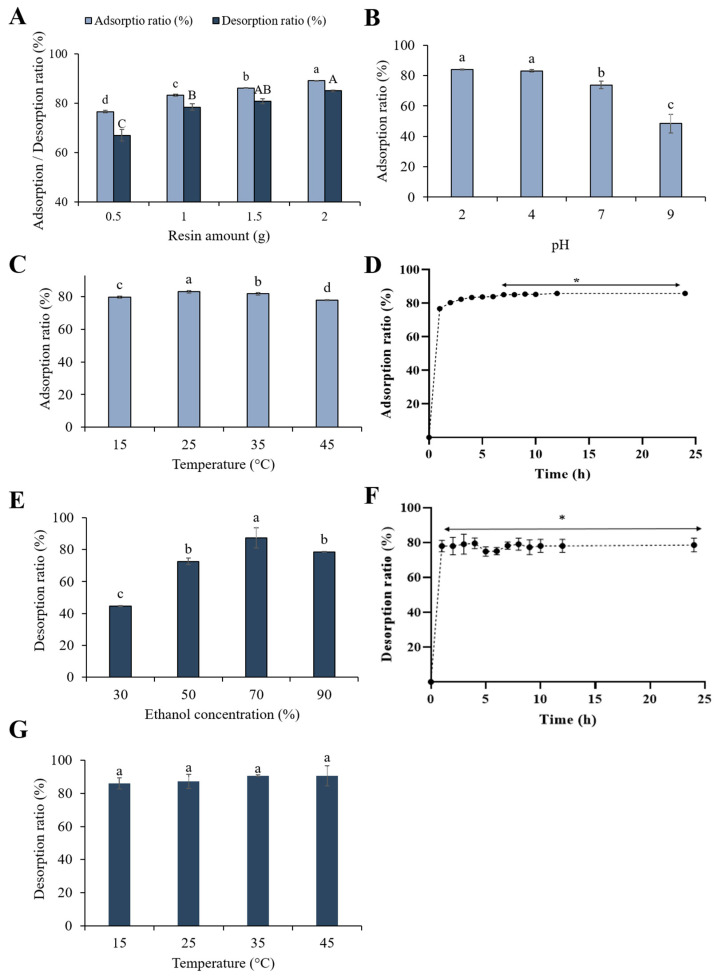
Single-factor experiments on the adsorption and desorption. (**A**) Effect of resin amounts; (**B**) Effect of pH; (**C**) Effect of the adsorption temperature; (**D**) Effect of adsorption time; (**E**) Effect of the ethanol concentration; (**F**) Effect of the desorption time; and (**G**) Effect of the adsorption temperature. * indicates plateau phase where equilibrium adsorption or desorption had been achieved. Error bars with different letters indicate a significant difference (*p* < 0.05) by Duncan’s multiple range test.

Figure 1A shows the influence of different XAD7HP amounts from 0.5 to 2 g on the adsorption and desorption ratios of polyphenol compounds from the CE. The lowest adsorption (76.70 ± 0.47%) and desorption ratio (67.03 ± 2.34%) were observed at 0.5 g, indicating that a small amount of resin adsorbed fewer polyphenol compounds [36]. Increasing the resin amount to 1 g resulted in an increased adsorption ratio of 83.39 ± 0.41% and a desorption ratio of 78.54 ± 1.35% (*p* < 0.05). Further increases in resin amount to 1.5 and 2 g also increased the adsorption ratios to 86.28 ± 0.14% and 89.17 ± 0.14%, respectively. As a result, with the increase in the resin amount, both the adsorption and desorption ratio increased significantly, with a more noticeable increase from 0.5 to 1 g than from 1 g to 2 g. Therefore, 1 g was selected as the optimal resin amount for further study. Similar result was reported by Wang et al. [23], who used macroporous resin for the recovery of polyphenols from distiller grains.

#### 3.2.2. Effect of pH

The pH of the crude extract (CE) solution is a key factor influencing the effectiveness of the adsorption process, as it significantly affects the ionization state of polyphenol compounds and their interaction with the resin [37]. As shown in Figure 1B, there was no significant difference observed between pH 2 (83.96 ± 0.42%) and 4 (83.08 ± 0.79%), (*p* > 0.05). However, as the pH levels increased from 4 to 9, the adsorption ratio notably decreased from 83.08 ± 0.79% to 48.43 ± 6.07% (*p* < 0.05). Our findings are consistent with a previous study by Xi et al. [37], who observed that the adsorption ratio of polyphenols from sweet potato (*Ipomoea batatas* L.) also significantly decreased with increasing pH. This can be attributed to the hydrogen-bonding interactions between the resin and polyphenol compounds. At high pH levels, the phenolic hydroxyl groups in polyphenol compounds dissociated to form H+ ions and formed anions, which reduces the adsorption capacity of polyphenols [37]. On the other hand, under acidic conditions, polyphenol compounds can be easily adsorbed by resin, due to increased hydrogen-bonding interactions resulting in a higher adsorption capacity [38]. As a result, pH 4 was selected for further experiment due to the pH of the freshly prepared CE solution was approximately 3.8–4.0.

#### 3.2.3. Effect of the Adsorption Temperature

The impact of temperature on the adsorption ratio of polyphenol compounds from the CE was assessed over a range from 15 °C to 45 °C. As shown in Figure 1C, the adsorption ratio increased significantly from 79.67 ± 0.65% to 83.08 ± 0.79% when the temperature was raised from 15 °C to 25 °C (*p* < 0.05). However, as the temperature was further increased from 25 °C to 45 °C, the adsorption ratio decreased from 83.08 ± 0.79% to 77.84 ± 0.34% (*p* < 0.05). This phenomenon could be attributed to the high temperatures reducing the adsorption efficiency by decreasing the stability of polyphenolic compounds or weakening interactions with the resin [39]. Interestingly, the previous studies [23,36] reported that temperature might not significantly influence the adsorption of some polyphenols, such as vanillin and distiller grains. However, it was observed that temperature was one of the significant variables determining the adsorption efficiency. Consequently, using an appropriate temperature could be crucial for effective adsorption. Thus, 25 °C was selected as the optimal temperature for subsequent experiments.

#### 3.2.4. Effect of Adsorption Time

The influence of time on the adsorption of polyphenol compounds in the CE was employed by using 1 g of XAD7HP resin, at a pH 4 of the CE solution, and 25 °C. As shown in Figure 1D, the adsorption ratio increased rapidly in the first hour and then slowly, reaching a plateau at around 7 h, with an adsorption ratio of 85.00 ± 0.43%. The rapid initial adsorption was due to the phenolic compound being easily accessible to mesopores on the surface of the resin particles. Meanwhile, the slower adsorption phase occurred because of mass transfer resistance within the particles [23]. As a result, 7 h was determined to be the optimal adsorption time.

Additionally, adsorption kinetics were analyzed using two models, the pseudo-first-order and pseudo-second-order models (Equations (4) and (5)), to understand the adsorption behavior. The results are presented in Table S1. The pseudo-first-order model primarily describes the initial phase of adsorption, while the pseudo-second-order model characterizes the entire adsorption process [18]. The kinetic analysis showed that the pseudo-first-order model had a correlation coefficient (R^2^) of 0.920. In contrast, the pseudo-second-order model demonstrated a higher R^2^ value of 0.981, indicating that the adsorption process followed a pseudo-second-order model. This finding aligns with similar studies on polyphenol adsorption [18,19].

#### 3.2.5. Effect of the Ethanol Concentration

The effect of different ethanol concentrations (30–90%) on the desorption of polyphenol compounds is shown in Figure 1E. When the ethanol concentration increased from 30% to 70%, the desorption ratio dramatically increased from 44.70 ± 0.37% to 87.31 ± 6.23% (*p* < 0.05). In contrast, further increasing the concentration to 90% significantly decreased the ratio from 87.31 ± 6.23% to 78.64 ± 0.20% (*p* < 0.05). A previous study reported that appropriate ethanol concentrations enhanced polyphenol desorption by modulating the polarity between the desorption solvent and the adsorbate [40]. An appropriate ethanol concentration can contribute to establishing matching polarity between ethanol and adsorbate, promoting competing intermolecular forces and facilitating their release into the solvent [41]. In this study, 70% ethanol solution might have induced competitive interaction between resin and polyphenol compounds, thereby boosting the desorption process [40]. However, at higher ethanol concentrations, other adsorbed substances from the resin may also be leached out, decreasing the desorption ratio [42]. This finding is consistent with the observation of Lianzhu et al. [19], who recovered phenolic compounds from Rabdosia serra using the same XAD7HP resin. As a result, 70% ethanol solution was used as the eluent in the desorption process.

#### 3.2.6. Effect of the Desorption Time

The effect of time on the desorption of polyphenol compounds from XAD7HP using 70% ethanol is shown in Figure 1F. The desorption ratio rapidly increased within the first hour, reaching approximately 85.81 ± 3.15%, followed by a plateau phase, indicating that equilibrium had been achieved. This result shows that most polyphenol compounds were effectively and quickly desorbed from the macroporous resin. Therefore, the optimal desorption time was 1 h.

#### 3.2.7. Effect of the Desorption Temperature

The effect of temperature on the desorption process was evaluated across a range from 15 °C to 45 °C. As shown in Figure 1G, there is no significant difference (*p* > 0.05) in desorption efficiency across these temperatures. The desorption ratio ranged from 85.22 ± 3.30% at 15 °C to 90.62 ± 6.04% at 45 °C. However, considering energy efficiency, 25 °C (87.10 ± 4.29%) was chosen as the optimal desorption temperature. This temperature-independent behavior differs from previous studies [43], which observed that the desorption ratio of polyphenols increased with temperature, reaching a maximum at 30 °C.

In summary, the optimum parameters for each factor were determined as follows: a resin amount of 1 g, a CE solution pH of 4, a temperature of 25 °C, an adsorption time of 7 h, an ethanol concentration of 70%, a desorption time of 1 h, and a temperature of 25 °C. Overall, these conditions achieved an 85.00% adsorption ratio, 87.10% desorption ratio, 69.75% recovery ratio, and 20.9% yield of the MRE from the CE.

### 3.3. Desorption Activation Energy

The activation energy of desorption was determined using the Arrhenius equation (Equation (6)). The desorption activation energy for polyphenols from the macroporous resin was 13 kJ/mol, which is slightly higher than the value reported by Gökmen [44]. This relatively low activation energy indicates that the desorption process rapidly releases polyphenols from the resin [45]. Additionally, this low activation energy highlights the energy efficiency of the desorption process, making it suitable for industrial applications.

### 3.4. Polyphenol Contents in the CE and MRE

**Figure 2:**
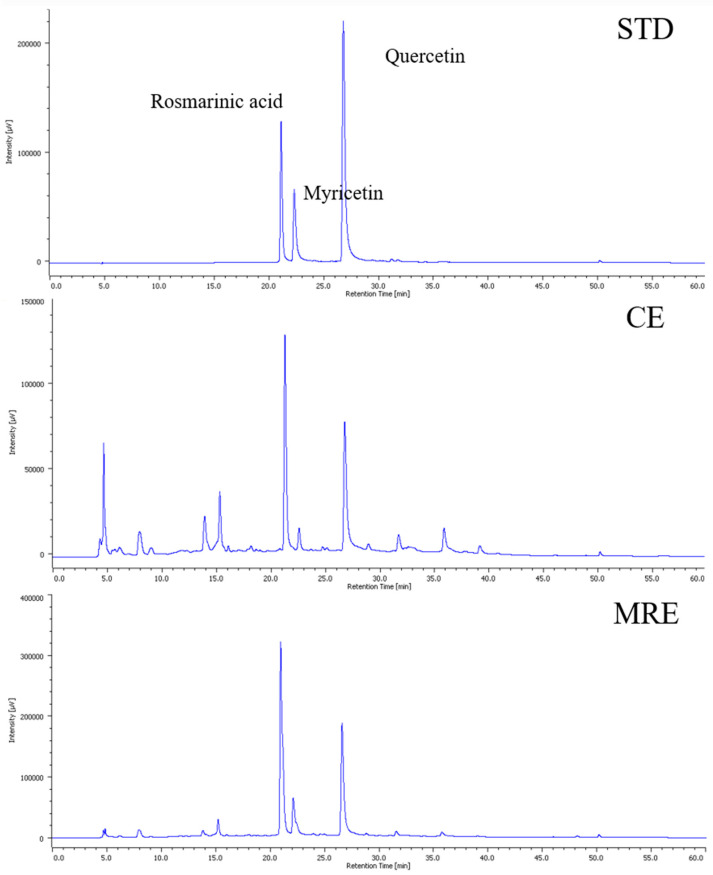
Chromatogram of standard (**top**), crude extract (**middle**), and macroporous resin-recovered extract (**bottom**) (condition: column C18 (250 × 4.6 mm, 5 μm), mobile phase: 1% aqueous acetic acid solution and acetonitrile, flow rate: 0.7 mL/min, UV detection at 254 nm).

Macroporous resin-recovered extract (MRE), obtained at the optimal adsorption and desorption conditions, was subjected to HPLC analysis to identify and quantify its polyphenol compounds in comparison with the CE. The obtained results are listed in Table 3 and Figure 2, respectively. The chromatogram of the CE (Figure 2top) exhibited various peaks, which could correspond to polyphenol compounds found in red onion peel, including quercetin, quercetin derivates, and phenolic acids [46]. Interestingly, it was identified that the major polyphenols after recovery were rosmarinic acid, quercetin, and myricetin (Figure 2middle,bottom). According to previous studies [2,9,47], polyphenols such as rosmarinic acid, quercetin, and myricetin were also identified in extracts from red onion peel using organic solvents like ethanol, methanol, toluene, and dichloromethane. As shown in Table 3, the optimal conditions efficiently recovered rosmarinic acid, quercetin, and myricetin from the CE. These polyphenols are known for their strong free radical scavenging activities and potential anti-inflammatory effects [48,49,50]. Notably, rosmarinic acid was identified as the predominant compound in the MRE. In summary, these results demonstrate approximately a sevenfold increase in the content of these polyphenol compounds after macroporous resin recovery. These findings suggest that macroporous resin effectively recover polyphenols and enhances a more antioxidant and anti-inflammatory potency of the MRE.

### 3.5. Structural Characterizations

#### 3.5.1. FT-IR

**Figure 3:**
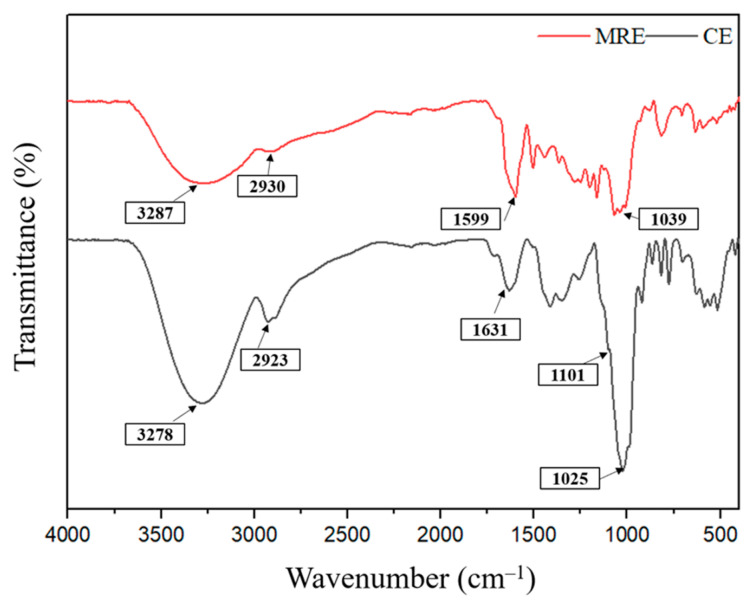
FT-IR spectra of the crude extract (CE) and macroporous resin-recovered extract (MRE) from red onion peel.

The FT-IR structural analysis was conducted to identify the structural difference between the CE and MRE (Figure 3). FT-IR spectra revealed that the CE and MRE have significantly different configurations, with distinct peak patterns, indicating that the macroporous resin recovery method altered the structural configuration of the CE. The spectrum of CE (black line) displayed a characteristic band at 3278 cm^−1^, which was attributed to the OH stretching vibration, indicating the presence of phenolic hydroxyl group. Peaks observed at 2923 cm^−1^ and 1631 cm^−1^ were associated with CH2 and CH3 stretching vibration and C=C stretching of the aromatic ring, respectively [51,52,53]. Additionally, the ‘shoulder’ peak at 1101 cm^−1^ and the sharp peak at 1025 cm^−1^ are assigned to the C-OH stretching band of oligosaccharide residue in the CE [54]. The FT-IR spectrum of the CE in this study was consistent with previous studies, suggesting that the CE from red onion peel contains polyphenol compounds and other substances [51,53]. The spectrum of the MRE (red line) also displayed characteristics bands at 3287 cm^−1^, 2930 cm^−1^, and 1599 cm^−1^, corresponding to the OH stretching vibrations, CH2 and CH3 stretching, and C=C stretching of the aromatic ring, respectively [55]. Notably, more prominent polyphenol peak patterns were observed in the MRE at 1500–1450 cm^−1^ (C-C-O stretching vibration) and 1300–1100 cm^−1^ (O-H bending vibrations) compared to the CE [55]. This alteration could be explained by the recovery of rosmarinic acid, quercetin, and myricetin, resulting from the recovery method [56,57,58]. Additionally, the peak at 1025 cm^−1^ in the CE shifted to 1039 cm^−1^ and decreased in intensity in the MRE, indicating an increase in the purity of polyphenol compounds [59]. Overall, given the differences between the CE and MRE, these results suggest that the macroporous resin effectively concentrate the polyphenol compounds from the CE.

#### 3.5.2. ^1^H-NMR Spectroscopy

**Figure 4:**
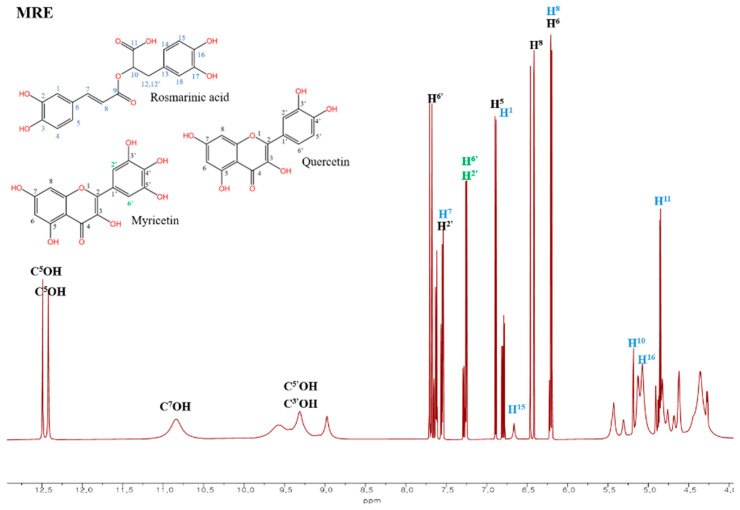
^1^H-NMR spectra of macroporous resin-recovered extract (MRE) from red onion peel.

The ^1^H-NMR spectra of the CE (Figure S2A), and MRE (Figure 4), along with the standard ¹H-NMR spectrum (Figure S2B) are presented. The ^1^H-NMR spectrum of the CE showed several signals between δ = 4.7–5.7 ppm, which correspond to the CH and CH2 groups in carbohydrate structures, which indicate the presence of carbohydrate content in the CE [60]. Also, characteristic aromatic proton signals of the polyphenols between δ = 6–7.5 ppm were detected [60]. These findings align with the FT-IR data, which also showed the presence of polysaccharides, highlighting that the CE contains non-polyphenol compounds. In contrast, the ^1^H-NMR spectrum of the MRE exhibited stronger aromatic proton signals between δ = 6.4–7.1 ppm compared to the CE. In addition, characteristic signals arising from the phenolic hydroxyl at δ = 8.82–12.51 ppm were observed in the MRE but were absent in the CE [56,58,61]. The increased signals observed in the MRE could be attributed to the recovery of polyphenols, including rosmarinic acid, quercetin, and myricetin. The assignment of the distinct proton signal for these polyphenols in the MRE is summarized in Figure S2B and Table S3. This phenomenon is further supported by the result of HPLC, where the increased polyphenol compounds were identified as major substances in the MRE, suggesting the macroporous resin recovery process effectively increase polyphenol contents in the MRE.

### 3.6. Antioxidant Properties

**Figure 5:**
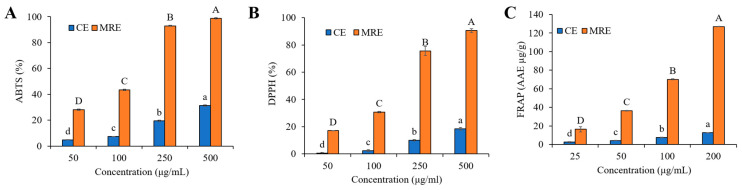
Antioxidant activity of the crude extract (CE) and macroporous resin-recovered extract (MRE) from red onion peel (**A**) ABTS assay; (**B**) DPPH assay; (**C**) FRAP assay. Different letters indicate a significant difference (*p* < 0.05) by Duncan’s multiple range test.

The ABTS·+ radical cation is generated by oxidizing ABTS, which typically remains stable until it reacts with hydrogen atom donors such as phenolic compounds. This reaction leads to a progressive change in the blue-green color of ABTS to a nearly colorless state, with the extent of the color change reflecting the antioxidant potential of the phenolic-containing samples [62]. As shown in Figure 5A, the ABTS scavenging activity of both the CE and MRE increased with concentration, from 50 μg/mL to 500 μg/mL. At 500 μg/mL, the ABTS scavenging ability of the MRE reached 98.75 ± 0.46%, while for the CE it was 31.39 ± 0.46% (*p* < 0.05). These results demonstrate that the MRE has significantly higher antioxidant activity compared to the CE. In contrast, onion peel extracts (*Allium cepa* L.) at 600 μg/mL exhibited a scavenging effect of approximately 60% for the ABTS radical [63], which is lower than the MRE.

In the DPPH, assay-antioxidants donate hydrogen atoms to the DPPH free radical, reducing it and resulting in a color change to yellow or colorless, depending on the degree of reduction [62]. Similar to the ABTS scavenging ability, the DPPH scavenging activity of both the CE and MRE increased with concentration (Figure 5B). At 500 μg/mL, the MRE demonstrated a DPPH scavenging ability of 90.60 ± 1.40%, which was significantly higher than the CE of 18.50 ± 1.10% (*p* < 0.05). Our result showed that the antioxidant activity of the MRE improved after the macroporous resin process, in line with previous studies [23], which also reported enhanced DPPH radical scavenging activity after macroporous resin recovery.

The FRAP assay measures the potential of antioxidants to reduce the Fe^3+^-TPTZ complex into a blue-colored Fe^2+^-TPTZ complex [64]. As shown in Figure 5C, both the CE and MRE showed concentration-dependent antioxidant activities, with the MRE demonstrating significantly higher activity. At 200 μg/mL, the FRAP value of the MRE reached 127.02 ± 0.24 μg AAE/g, while the CE achieved only 12.65 ± 0.04 μg AAE/g (*p* < 0.05). This reducing ability is likely due to the phenolic content in the samples, as phenolic compounds act as reducing agents by donating hydrogen atoms and quenching singlet oxygen [65]. Similarly, a previous study [66] reported a positive correlation between the FRAP value and the total polyphenol content of onion peel extract.

The improved antioxidant activity of the MRE can be attributed to the efficient recovery of polyphenols from the CE using macroporous resin. As confirmed in Section 3.3, the increased presence of antioxidant polyphenols such as rosmarinic acid, quercetin, and myricetin in the MRE might have contributed to its radical scavenging ability [48,49,50]. Additionally, several studies have demonstrated a dose-dependent relationship between polyphenol content and antioxidant capacity [67]. Overall, these findings suggest that the resin recovery method significantly enhanced the antioxidant activity of the CE, highlighting the potential advantages of this process.

### 3.7. In Vitro Anti-Inflammatory Activities

Nitric oxide and reactive oxygen species (ROS) are the major oxidative stress mediators produced by macrophages, which induce dysfunction of not only macrophages but also surrounding normal cells [68,69]. The anti-inflammatory properties of the CE and MRE were confirmed by identifying the reduction in nitric oxide synthesis and ROS production in LPS-induced activated RAW264.7 cells.

**Figure 6:**
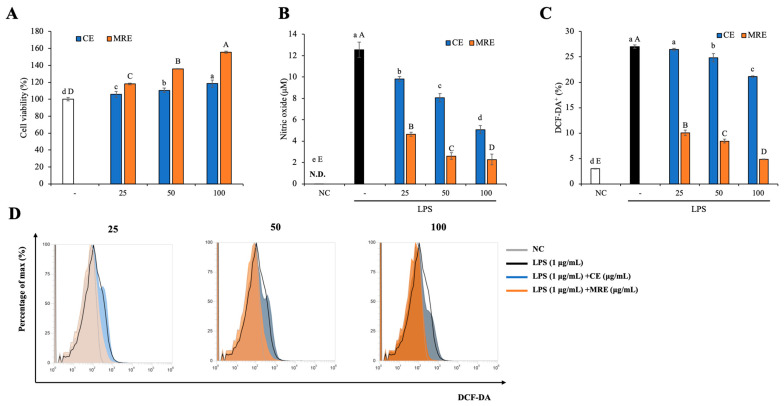
Anti-inflammatory activities of CE and MRE in LPS-induced activated RAW264.7 cells. (**A**) RAW264.7 cells were treated with various concentrations of CE or MRE for 24 h, and the cell viability was analyzed by CCK-8 assay. (**B**–**D**). Cells were pretreated with various concentrations of CE or MRE for 1 h, then cotreated with LPS for 24 h. The nitric oxide synthesis was analyzed by Griess reagent, and the intracellular ROS production was measured by DCF-DA assay using flow cytometry. The data are presented as mean ± SD. Different letters indicate statistically significant different. If the measurement value was too low and there was no result, it was marked as ‘N.D (Not detected)’.

Figure 6A presents the effects of the CE and MRE on RAW264.7 cells at the tested concentrations (25–100 μg/mL). Both the CE and MRE showed increased cell viability (105.76–118.42% and 118.04–155.61%) compared to the nontreatment group (100.00 ± 2.11%). This result reveals that the CE and MRE are non-cytotoxic, as well as promote RAW264.7 cell proliferation.

Figure 6B presents the anti-inflammatory activities of the CE and MRE on the LPS-induced activated RAW264.7 cells. Increased nitric oxide synthesis was observed by LPS treatment (12.54 ± 0.72 μM), which was significantly reduced in a dose-dependent manner by the CE (9.81–5.07 μM) and MRE (4.63–2.28 μM) treatment. Likewise, an increase in DCF-DA+ area was observed by LPS treatment (27.00 ± 0.36%), which was significantly decreased dose-dependently by the CE (26.44–21.10%) and MRE (10.06–4.86%), suggesting that the CE and MRE mitigate LPS-induced intracellular ROS production (Figure 6C,D and Figure S3). These results suggest that the CE and MRE alleviate LPS-induced inflammation in RAW264.7 cells, and it is noteworthy that the MRE showed superior anti-inflammation activities compared to the CE. The enhanced anti-inflammatory activities of the MRE can be mainly attributed to its increased rosmarinic acid, myricetin, and quercetin contents, which have been reported to have anti-inflammatory activities in LPS-induced activated RAW264.7 cells [70,71,72].

## 4. Conclusions

In this study, polyphenol extracts were recovered from red onion peel, an agricultural waste, using macroporous resin. Among the four resin types examined, XAD7HP was most efficient, demonstrating high desorption and recovery ratios. Optimal adsorption parameters were determined through single-factor experiments: 1 g of resin, solution pH of 4 at 25 °C, and adsorption time of 7 h. Optimal desorption parameters were 70% ethanol for 1 h at 25 °C. Kinetic studies showed that the adsorption process was best described by pseudo-second-order kinetics. The Arrhenius equation demonstrated a low activation energy requirement (13 kJ/mol) for desorption, highlighting the energy efficiency of the recovery process. The recovered extract (MRE) was characterized using HPLC, FT-IR, and ¹H-NMR analyses, confirming the presence of polyphenol compounds including rosmarinic acid, quercetin, and myricetin, with concentrations approximately sevenfold higher compared to the crude extract (CE). Consequently, the MRE exhibited notably enhanced antioxidant activity, demonstrated through ABTS, DPPH, and FRAP assays. Moreover, the MRE showed improved anti-inflammatory effects, including inhibition of nitric oxide synthesis and reduction in reactive oxygen species (ROS) production in LPS-induced RAW264.7 cells. This study highlights that red onion peel is a promising biowaste rich in bioactive compounds, which can be effectively recovered through the macroporous resin process for enhanced antioxidant and anti-inflammatory properties, applicable in the food, cosmetic, and pharmaceutical industries. Further optimization studies and in-depth investigations of dynamic adsorption and desorption mechanisms are necessary for industrial-scale applications.

## Figures and Tables

**Table 1:** Physical properties of the four types of macroporous resins assessed in this study.

Resins	SP850	XAD16N	XAD7HP	XAD2
	Physicochemical properties			
Material	Styrene-divinylbenzene	Styrene-divinylbenzene	Acrylic	Styrene-divinylbenzene
Polarity	Non-polar	Non-polar	Medium polarity	Non-polar
Porosity (mL/g)	<1.2	<0.55	0.5	<0.65
Particle diameter (mm)	0.25–0.85	0.56–0.71	0.56–0.71	0.25–0.84
Surface area (m^2^/g)	<1000	800	380	<300
Average pore size (Å)	38	200	300–400	90

**Table 2:** Adsorption, desorption, and recovery ratio (%) of four different resins.

Resins	SP850	XAD16N	XAD7HP	XAD2
Adsorption/desorption/recovery properties				
Adsorption ratio (%)	82.39 ± 0.95 a	82.15 ± 0.24 a	81.34 ± 0.61 a	76.20 ± 0.24 b
Desorption ratio (%)	73.16 ± 1.18 ab	72.34 ± 1.75 ab	78.54 ± 1.35 a	65.69 ± 7.8 b
Recovery ratio (%)	64.49 ± 0.53 b	62.75 ± 0.43 c	68.86 ± 0.9 a	58.31 ± 1.41 d

Values are the mean ± SD (n = 3). Different letters in the same low indicate significant differences (*p* < 0.05).

**Table 3:** Identified polyphenol compounds by HPLC and Total Phenolic Content (TPC) by UV-visible spectroscopy, and their content (mg/g) in the crude extract (CE) and macroporous resin-recovered extract (MRE) from red onion peel.

	Rosmarinic Acid (mg/g)	Myricetin (mg/g)	Quercetin (mg/g)	TPC (mg GAE/g)
CE	54.26 ± 0.3	10.44 ± 0.31	16.4 ± 0.48	38.65 ± 1.50
MRE	402.76 ± 15.1	69.88 ± 6.83	112.85 ± 2.46	188.52 ± 1.53

Values are the mean ± SD (n = 3).

## Data Availability

The data presented in this study are available on request from the corresponding author.

## Supplementary Materials

The following supporting information can be downloaded at: https://www.mdpi.com/article/10.3390/antiox14020145/s1, Table S1: Adsorption kinetic parameters of phenolic compounds; Table S2: Desorption kinetic parameters for desorption activation energy; Table S3: ^1^H-NMR chemical shifts of the polyphenol compounds detected in the macroporous resin-recovered extract (MRE) from red onion peel in DMSO-d6 at 298 K; Figure S1. Arrhenius plot of desorption rate constants; Figure 2: (A) ^1^H-NMR spectra of crude extract (CE) from red onion peel; (B) ^1^H-NMR spectra of mix standards; rosmarinic acid, myricetin and quercetin (1 mg/mL); Figure S3: Flow cytometry gating strategy (A) and actual results of DCF-DA assay (B) on the LPS-induced activated RAW264.7 cells according to the CE or MRE treatment.

## Abbreviations

The following abbreviations are used in this manuscript:

CE	Crude extract
MRE	Macroporus resin-recovered extract
STD	Standard
