# Supplementary material for: Macroporous Resin Recovery of Antioxidant Polyphenol Compounds from Red Onion (Allium cepa L.) Peel
Source: Antioxidants (Basel). 2025 Jan 26;14(2):145. doi: 10.3390/antiox14020145 (PMC11852097; doi:10.3390/antiox14020145)
Supplement: Supplementary file 1 [file antioxidants-14-00145-s001.zip › antioxidants-3418047-supplementary.pdf]

Table S1. Adsorption kinetic parameters of phenolic compounds

**Pseudo-first-order model**		**Pseudo-second-order model**	
k_1	R^2	k_2	R^2
0.531	0.920	1.226	0.981

Table S2. Desorption kinetic parameters for desorption activation energy.

**Temperature (°C)**	15	25	35	45
***k* (min⁻¹)**	0.0021	0.0025	0.0032	0.0035
**E_d (J/mol)**		13344.6		

Table S3. ^{1}H-NMR chemical shifts of the polyphenol compounds detected in macroporous resin-recovered extract (MRE) from red onion peel in DMSO-d6 at 298 K

		Compound	
no	**Rosmarinic acid**	**Quercetin**	**Myricetin**
1	4.86 (dd, H^{11})	6.20 (m, H^6)	6.20 (m, H^6)
2	5,07 (s, H^{16})	6.41 (d, H^8)	6.41 (d, H^8)
3	5,19 (d, H^{10})	6.89 (d, $H^{5'}$)	6.89 (d, $H^{5'}$)
4	6.20 (m, H^8)	7.55 (m, $H^{2'}$)	7.27 (m, $H^{2'}$, $H^{6'}$)
5	6.66 (s, H^{15})	7.65 (m, $H^{6'}$)	9.31 (s, $C^{3'}$OH, $C^{5'}$OH)
6	6.8 (dd, H^1)	9.31 (s, $C^{3'}$OH)	10.84 (s, C^7OH)
7	7.55 (m, H^7)	10.84 (s, C^7OH)	12.42 (s, C^5OH)
8	-	12.49 (s, C^5OH)	-

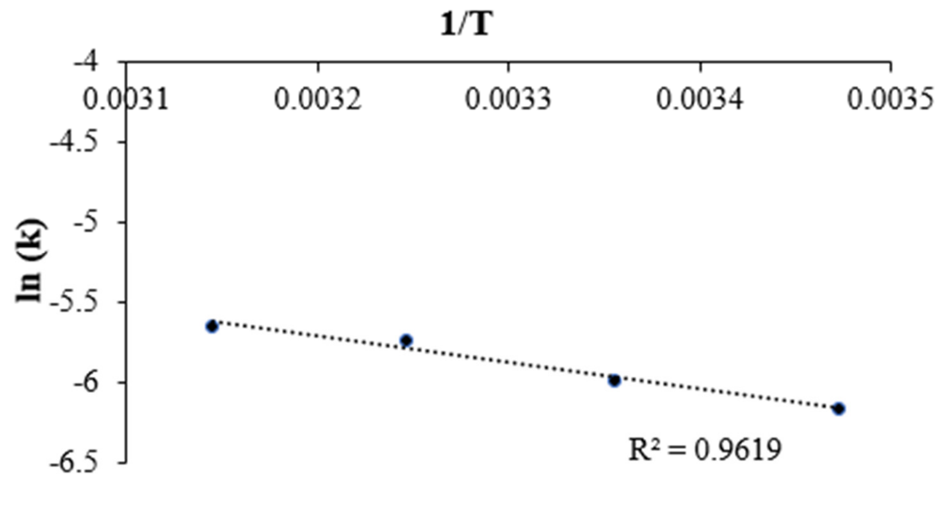

Figure S1. Arrhenius plot of desorption rate constants

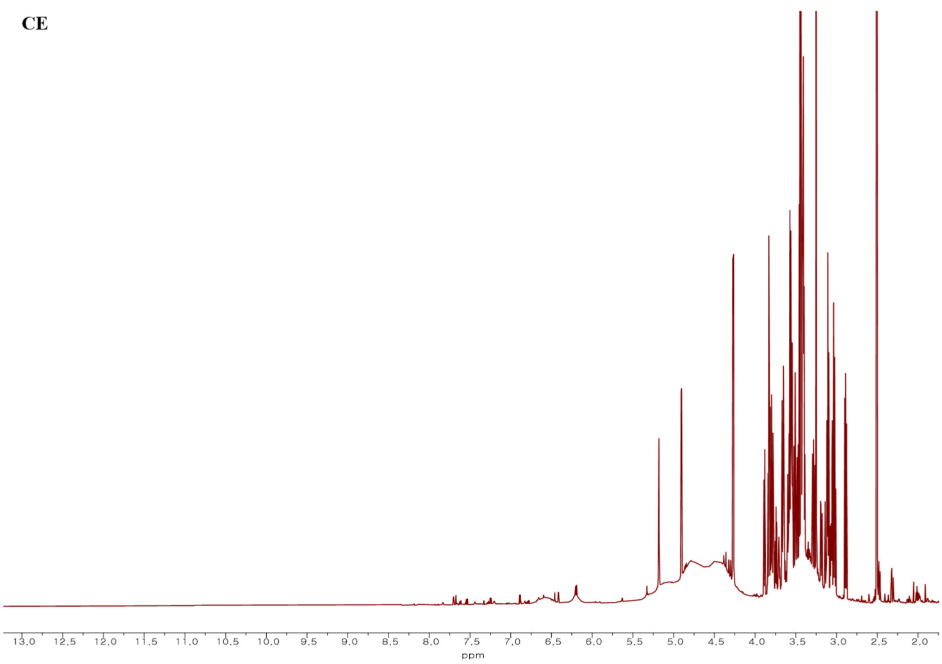

Figure S2A. ^{1}H-NMR spectra of crude extract (CE) from red onion peel.

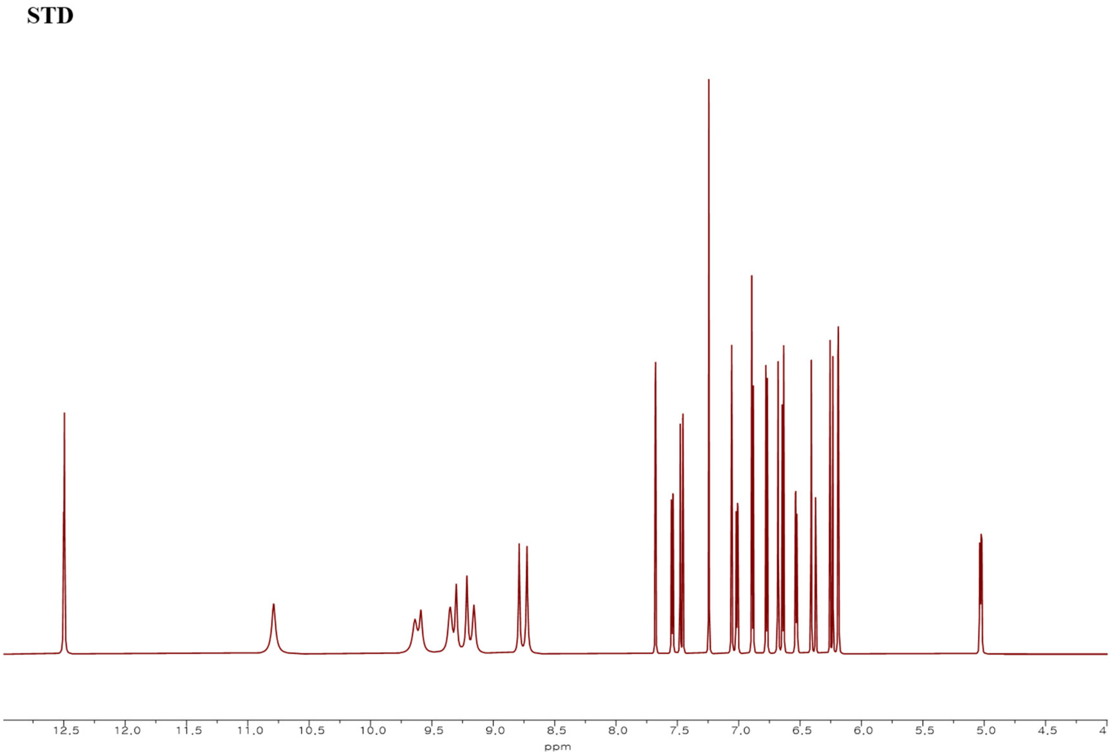

Figure S2B. ^{1}H-NMR spectra of mix standards; rosmarinic acid, myricetin and quercetin (1mg/ml).

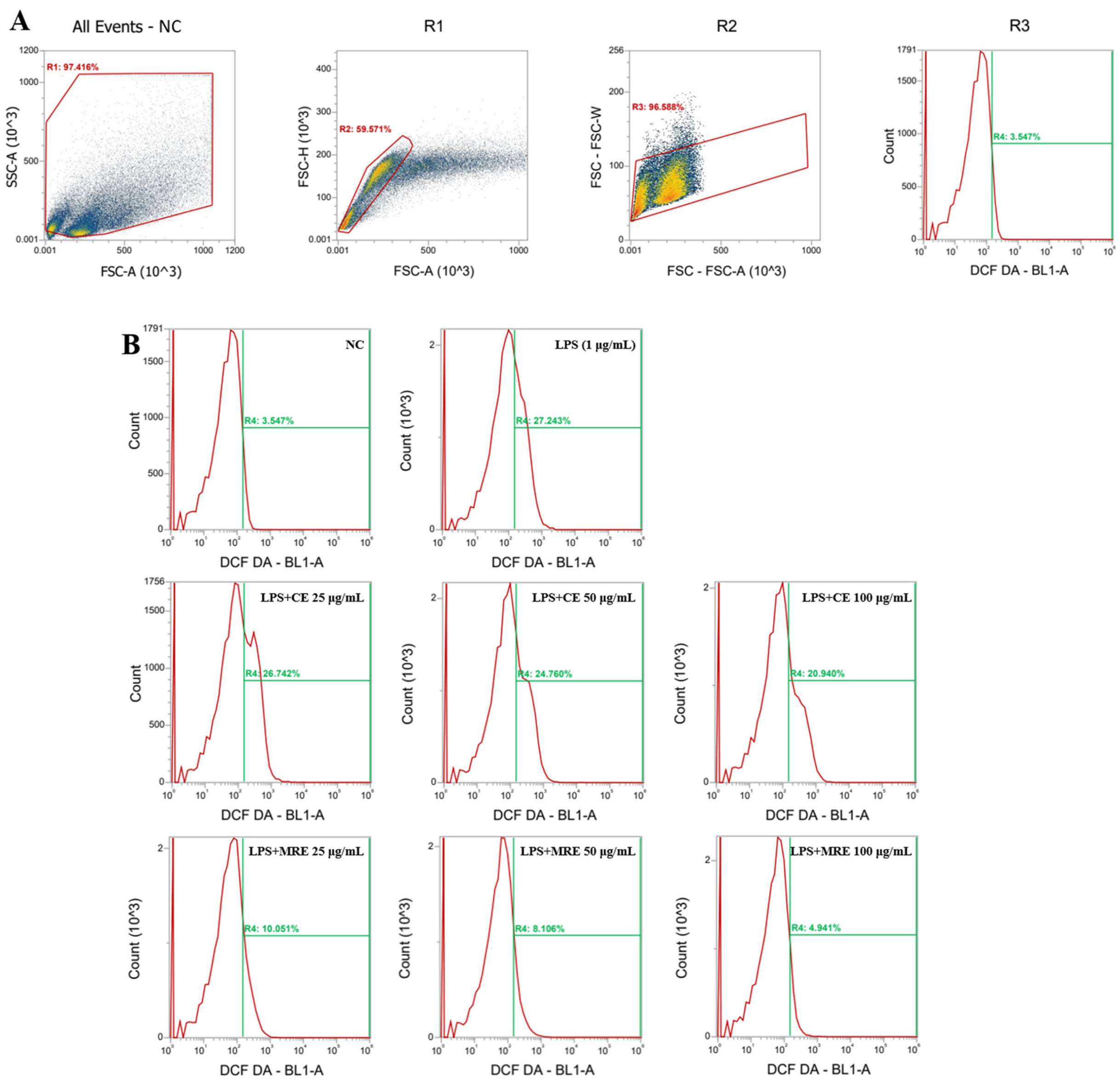

Figure S3. Flow cytometry gating strategy (A) and actual results of DCF-DA assay (B) on the LPS-induced activated RAW264.7 cells according to CE or MRE treatment.
